# Overexpression of Snail is associated with lymph node metastasis and poor prognosis in patients with gastric cancer

**DOI:** 10.1186/1471-2407-12-521

**Published:** 2012-11-14

**Authors:** Na Ri Shin, Eun Hui Jeong, Chang In Choi, Hyun Jung Moon, Chae Hwa Kwon, In Sun Chu, Gwang Ha Kim, Tae Yong Jeon, Dae Hwan Kim, Jae Hyuk Lee, Do Youn Park

**Affiliations:** 1 Department of Pathology, Pusan National University Hospital and Pusan National University School of Medicine, 1-10 Ami-Dong, Seo-Gu, Busan, 602-739, South Korea; 2 Department of Internal Medicine, Pusan National University Hospital and Pusan National University School of Medicine, Busan, South Korea; 3 Department of Surgery, Pusan National University Hospital and Pusan National University School of Medicine, Busan, South Korea; 4 BioMedical Research Institute, Pusan National University Hospital, Busan, South Korea; 5 Department of Pathology, Cheonam National University, Gwangju, South Korea; 6 Korean Bioinformation Center, Korea Research Institute of Bioscience and Biotechnology, Daejeon, South Korea

**Keywords:** Stomach, Adenocarcinoma, Snail, Lymph node metastasis, Survival

## Abstract

**Background:**

Epithelial–mesenchymal transition (EMT) plays a significant role in tumor progression and invasion. Snail is a known regulator of EMT in various malignant tumors. This study investigated the role of Snail in gastric cancer.

**Methods:**

We examined the effects of silenced or overexpressed Snail using lenti-viral constructs in gastric cancer cells. Immunohistochemical analysis of tissue microarrays from 314 patients with gastric adenocarcinoma (GC) was used to determine Snail’s clinicopathological and prognostic significance. Differential gene expression in 45 GC specimens with Snail overexpression was investigated using cDNA microarray analysis.

**Results:**

Silencing of Snail by shRNA decreased invasion and migration in GC cell lines. Conversely, Snail overexpression increased invasion and migration of gastric cancer cells, in line with increased VEGF and MMP11. Snail overexpression (≥75% positive nuclear staining) was also significantly associated with tumor progression (*P* < 0.001), lymph node metastases (*P* = 0.002), lymphovascular invasion (*P* = 0.002), and perineural invasion (*P* = 0.002) in the 314 GC patients, and with shorter survival (*P* = 0.023). cDNA microarray analysis revealed 213 differentially expressed genes in GC tissues with Snail overexpression, including genes related to metastasis and invasion.

**Conclusion:**

Snail significantly affects invasiveness/migratory ability of GCs, and may also be used as a predictive biomarker for prognosis or aggressiveness of GCs.

## Background

Epithelial–mesenchymal transition (EMT), a developmental process whereby epithelial cells reduce intercellular adhesion and acquire myofibroblastic features, is critical to tumor progression [1-3]. During EMT, significant changes occur, including downregulation of epithelial markers such as E-cadherin, translocation of β-catenin (i.e., dissociation of membranous β-catenin and translocation into the nuclear compartment), and upregulation of mesenchymal markers such as vimentin and N-cadherin [3-6]. EMT is induced by repression of E-cadherin expression by EMT regulators such as Snail, Slug, and Twist. The Snail family of zinc-finger transcriptional repressors directly represses E-cadherin *in vitro* and *in vivo* via an interaction between their COOH-terminal region and the 5^′^-CACCTG-3^′^ sequence in the E-cadherin promoter [7-9]. Snail is reportedly important in several carcinomas, including non-small cell lung carcinomas, ovarian carcinomas, urothelial carcinomas, and hepatocellular carcinoma [10-13]. Studies have also used immunohistochemical analyses to show the clinical significance of Snail overexpression in gastric adenocarcinoma (GC) [14,15]. However, few reports on the roles of Snail in GC have included clinicopathological, prognostic, and functional *in vitro* analyses as well as gene expression results. We therefore evaluated Snail’s effect on invasiveness/migratory ability in gastric cancer cell lines, and also investigated the possibility of Snail being used as a predictive marker for evaluating poor prognosis or tumor aggressiveness in GC patients. We also evaluated the gene expression pattern in 45 GC tissues with Snail overexpression, using cDNA microarrays.

## Methods

### shRNA lentivirus-mediated silencing and overexpression of Snail in gastric cancer cells

Human gastric cancer cell lines SNU216 and SNU484 were obtained from Korean Cell Line Bank (KCLB) and were authenticated by DNA profiling. These cells cultured in RPMI1640 medium with 10% fetal bovine serum (FBS), 100 U/ml penicillin, and 100 μg/ml streptomycin (hyClone, Ogden, UT). All cells were maintained at 37°C in 5% CO_2_. Lentiviral-based RNA knockdown and overexpression were used for silencing and overexpression of Snail. Lentiviruses expressing either non-target or *Snail*-targeted shRNAs were used for silencing; a PLKO lentiviral vector targeting *Snail* or an empty PLKO vector were used for overexpression of Snail in the SNU216 and SNU484 cells. Lentivirus stocks were produced using the Virapower™ lentiviral packaging mix using the 293FT cell line according to the manufacturer’s protocol (Invitrogen, Carlsbad, CA). SNU216 and SNU484 cells grown to 50% confluence were incubated for 24 h in a 1:1 dilution of virus:media with 5 μg/ml Polybrene. After a 24-h recovery period in complete media without virus, polyclonal stable cell lines were selected and maintained in media containing 5 μg/ml puromycin. Silencing or overexpression of Snail was determined by RT-PCR and western blotting.

### Real time RT-PCR analysis of *VEGF*, *MMP11*, and *Snail* in gastric cancer cells

Total cellular RNA was extracted using the TRIzol method (Sigma-Aldrich, St Louis, MO, USA). For RT-PCR analysis, 2-μg aliquots of RNA were subjected to cDNA synthesis with 200 U of MMLV reverse transcriptase and 0.5 μg of oligo(dT)-15 primer (Promega, Madison, WI, USA). Quantitative real-time PCR was performed with the Rotor-Gene™ System (QIAGEN, Hilden, Germany) using AccuPower 2× Greenstar qPCR Master Mix (Bioneer, Daejeon, Korea). cDNA in 1 μl of the reaction mixture was amplified with 0.5 U of GoTaq DNA polymerase (Promega) and 10 pmol each of the following sense and antisense primers: *GAPDH* 5^′^-TCCATGACAACTTTGGTATCG-3^′^, 5^′^-TGTAGCCAAATTCGTTGTCA-3^′^; *Snail* 5^′^-CTTCCTCTCCATACCTG-3^′^, 5^′^-CATAGTTAGTCACACCTCGT-3^′^; *VEGF* 5^′^-TTGCTGCTCTACCTCCACCA-3^′^, 5^′^-GCACACAGGATGGCTTGAA-3^′^; *MMP11* 5^′^-CTTGGCTGCTGTTGTGTGCT-3^′^, 5-AGGTATGGAGCGATGTGACG-3^′^. The thermal cycling profile was: denaturation for 30 s at 95°C, annealing for 30 s at 52°C (depending on the primers used), and extension for 30 s at 72°C. For semi-quantitative assessment of expression levels, 30 cycles were used for each PCR reaction. PCR products were size-fractionated on 1.0% ethidium bromide/agarose gels and quantified under UV transillumination. The threshold cycle (CT) is defined as the fractional cycle number at which the fluorescence passes a fixed threshold above baseline. Relative gene expression was quantified using the average CT value for each triplicate sample minus the average triplicate CT value for *GAPDH*. Differences between the control (empty vector) and experiment groups (infected with the lentivirus) were calculated using the formula 2 ^– ([△CT Lenti] – [△CT control])^ and expressed as a fold change in expression according to the comparative threshold cycle method (2–^△△CT^) [16].

### Western blotting

Cells were harvested and disrupted in lysis buffer (1% Triton X-100, 1mM EGTA, 1mM EDTA, 10mM Tris–HCl, pH 7.4 and protease inhibitors). Cell debris was removed by centrifugation at 10,000 × *g* for 10 min at 4°C. The resulting supernatants were resolved on a 12% SDS-PAGE under denatured reducing conditions and transferred to nitrocellulose membranes. The membranes were blocked with 5% non-fat dried milk at room temperature for 30 min and incubated with primary antibodies. The membranes were washed and incubated with horseradish peroxidase-conjugated secondary antibody. The signal was visualized using an enhanced chemiluminescence (Amersham, Buckinghamshire, UK).

### Cell migration and Matrigel invasion assay

Gastric cancer cells were harvested with 0.05% trypsin containing 0.02% EDTA (Sigma-Aldrich), and suspended in RPMI at a concentration of 3 × 10^3^ cells/well. Membrane filters (pore size: 8 μm) in disposable 96-well chemotaxis chambers (Neuro Probe, Gaithersburg, MD) were pre-coated for 4 h with 5 mg/ml fibronectin at room temperature. Aliquots (50 μl/well) of the cell suspension were loaded into the upper chambers, and 1% FBS was loaded into the lower chamber. After 24-h incubation, non-migrating cells were removed from the upper chamber with a cotton swab; cells present on the lower surface of the insert were stained with Hoechst33342 (Sigma-Aldrich). Invasive cells were counted under a fluorescence microscope at × 10 magnification.

For the Matrigel invasion assay, 3 × 104 cells/well were seeded in the upper chamber, which was coated with Matrigel (5 mg/ml in cold medium, BD Transduction Laboratories, Franklin Lakes, NJ, USA), and serum-free medium containing 1% FBS or control vehicle was added to the lower chamber. After 24-h incubation, non-migrating cells were removed from the upper chamber with a cotton swab, and cells present on the lower surface of the insert were stained with Hoechst33342 (Sigma-Aldrich). Invasive cells were then counted under a fluorescence microscope at × 10 magnification.

### Tissue microarrays, immunohistochemistry, and interpretation of results

A semi-automated tissue arrayer (Beecher Instruments, WI, USA) was used to construct the tissue microarrays. We obtained 3 tissue cores, each 0.6 mm in diameter, from tumor blocks taken from GC patients. Cores were not collected from the more invasive frontal or central areas of the tumors. Slides were baked at 60°C for 30 min, deparaffinized with xylene, and then rehydrated. The sections were subsequently submerged in citrate antigen retrieval buffer, microwaved for antigen retrieval, treated with 3% hydrogen peroxide in methanol to quench endogenous peroxidase activity, and then incubated with 1% bovine serum albumin to block non-specific binding. Thereafter, the sections were incubated with rabbit anti-Snail (Abcam, UK) overnight at 4°C. Normal rabbit serum was used as a negative control. After washing, tissue sections were treated with secondary antibody, counterstained with hematoxylin, dehydrated, and mounted. At least 500 tumor cells were counted. The percentage of cells with Snail^+^ nuclei was expressed relative to the total number of tumor cells counted. Nuclear expression of Snail was graded by classifying the extent of positive nuclear staining as ≤50%, 50–75%, or ≥75%.

### Clinicopathological and survival analysis of gastric cancer patients

We studied a cohort of 314 GC patients who each underwent a gastrostomy with lymph node dissection at Pusan National University Hospital (PNUH) between 2005 and 2007. The group comprised 218 men and 96 women with a mean age of 58.3 years (range, 25–83 years). Standard formalin-fixed and paraffin-embedded sections were obtained from the Department of Pathology, PNUH, and the National Biobank of Korea, PNUH. The study was approved by the Institutional Review Board. None of the patients received preoperative radiotherapy and/or chemotherapy. Adjuvant chemotherapy based on 5-FU was administered on patients with stages II, III and IV after curative resection. We assessed several clinicopathological factors according to the Korean Standardized Pathology Report for Gastric Cancer, the Japanese Classification of Gastric Carcinoma (3^rd^ English edition), and the American Joint Committee on Cancer Staging Manual (7^th^ edition), including tumor site, gross appearance and size, depth of invasion, histological classification (i.e., intestinal or diffuse), and lymphovascular invasion [17-19]. Clinical outcome for each patient was followed from the date of surgery to the date of death or March 1, 2012. Follow-up periods ranged from approximately 1 to 81.5 months (average, 51.4 months). Cases lost to follow-up or death from any cause other than gastric cancer were censored from the survival rate analysis. Clinicopathological features were analyzed using Student’s *t*-test, the χ^2^ test, or Fisher’s exact test to test for differences in Snail expression. Cumulative survival plots were obtained using the Kaplan–Meier method, and significance was compared using the log-rank test. Prognostic factors were identified using the Cox regression stepwise method (proportional hazard model), adjusted for the patients’ age, gender, tumor site, morphologic type (intestinal versus diffuse). Statistical significance was set at *P* < 0.05. Statistical calculations were performed with SPSS version 10.0 for Windows (SPSS Inc., Chicago, IL, USA).

### cDNA microarray analysis of GC tissues based on Snail overexpression

A total of 45 fresh GC tissues were obtained from the National Biobank of Korea, PNUH, and CNUH; approval was obtained from their institutional review boards. Total RNA was extracted from the fresh-frozen tissues using a mirVana RNA Isolation kit (Ambion Inc., Austin, TX). Five hundred nanograms of total RNA was used for cDNA synthesis, followed by an amplification/labeling step (*in vitro* transcription) using the Illumina TotalPrep RNA Amplification kit (Ambion) to synthesize biotin-labeled cRNA. cRNA concentrations were measured by the RiboGreen method (Quant-iT RiboGreen RNA assay kit; Invitrogen-Molecular Probes, ON, Canada) using a Victor3 spectrophotometer (PerkinElmer, CT), and cRNA quality was determined on a 1% agarose gel. Labeled, amplified material (1500 ng per array) was hybridized to Illumina HumanHT-12 BeadChips v4.0, according to manufacturer’s instructions (Illumina, San Diego, CA). Array signals were developed by streptavidin-Cy3. Arrays were scanned with an Illumina iScan system. The microarray data were normalized using the quantile normalization method in Illumina BeadStudio software. The expression level of each gene was transformed into a log^2^ base before further analysis. Excel was primarily used for statistical analyses. Gene expression differences were considered statistically significant if *P* < 0.05; all tests were 2-tailed. Cluster analyses were performed using Cluster and Treeview [20]. The gene ontology (GO) program (http://david.abcc.ncifcrf.gov/) was used to categorize genes into subgroups based on biological function. Fisher’s exact test was used to determine whether the proportions of genes in each category differed by group. GC tissues were further divided into those with higher (≥75%) and lower (<75%) levels of Snail expression; differential gene expression between the groups was identified. Primary microarray data are available in NCBI’s GEO (Gene Expression Omnibus) database (http://www.ncbi.nlm.nih.gov/geo/query/acc.cgi?acc=GSE38024).

## Results

### Regulation of migration and invasion of gastric cancer cells by Snail

**Figure 1:**
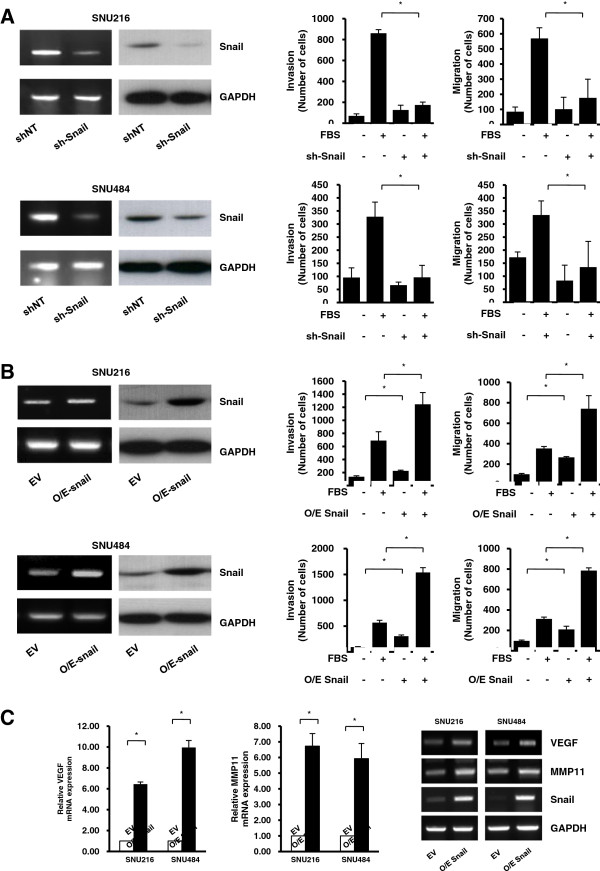
**Role of Snail in invasion and migration of gastric cancer cell lines. A**. SNU216 (upper panel) and SNU484 (lower panel) cells were infected with lentiviruses expressing either non-target shRNA (*shNT*) or *Snail* shRNA on day 0, and then harvested on day 7 post-infection. *Snail* knockdown was determined by RT-PCR and western blotting; stable cell lines were generated for each of the cell lines (sh-Snail). Silencing of *Snail* in SNU216 and SNU484 cells induced decreased migration and invasion. **B**. SNU216 (upper panel) and SNU484 (lower panel) cells were infected with lentiviruses expressing either a lentiviral PLKO vector targeting *Snail* or an empty PLKO vector (EV) on day 0, and then harvested on day 7 post-infection. The overexpression of Snail was determined by RT-PCR and western blotting; stable cell line was generated for each of the cell lines (O/E-snail). Snail overexpression in SNU216 and SNU484 cells induced increased migration and invasion. **C**. Snail overexpression induced increased mRNA expression of *VEGF* and *MMP11* in SNU216 and SNU484 cells in real-time RT-PCR analysis. Lower panel indicates representative RT-PCR figures for *VEGF*, *MMP11*, *Snail*, and *GAPDH*. Data show the mean ± SE of at least 3 independent experiments. * indicates *P* < 0.05 by Student’s *t*-test.

Lentiviral-based RNA knockdown and overexpression approaches were used to determine Snail’s role in invasion and migration of gastric cancer cell lines. SNU216 and SNU484 cells used in this study are established gastric adenocarcinoma cell lines from Korean patients. These cells were infected with a lentivirus expressing either non-target or *Snail*-targeted shRNAs for silencing. A PLKO lentiviral vector that targeted *Snail* and an empty PLKO vector were used to induce Snail overexpression in SNU216 and SNU484 cells. Polyclonal stable cell lines were selected using puromycin. *Snail* expression was determined by RT-PCR and western blotting; stable *Snail* knockdown (sh-Snail) and Snail overexpression cell lines (OE-Snail) were obtained (Figure 1).

To determine Snail’s roles in gastric cancer cell invasion, we measured chemotactic invasion by the cells using the Transwell system with filters pre-coated with Matrigel. To measure migration of gastric cancer cells, we assayed cell migration using a Boyden chamber apparatus. Silencing of *Snail* by shRNA induced decreased migration and invasion of SNU216 and SNU484 cells, as shown in Figure 1A. In contrast to the *Snail* silencing results, overexpression of Snail induced increased migration and invasion of SNU216 and SNU484 cells, as shown in Figure 1B. Overexpression of Snail was also associated with increased VEGF and MMP11 (Figure 1C).

### Effect of Snail overexpression on tumor aggressiveness and GC patient survival

**Figure 2:**
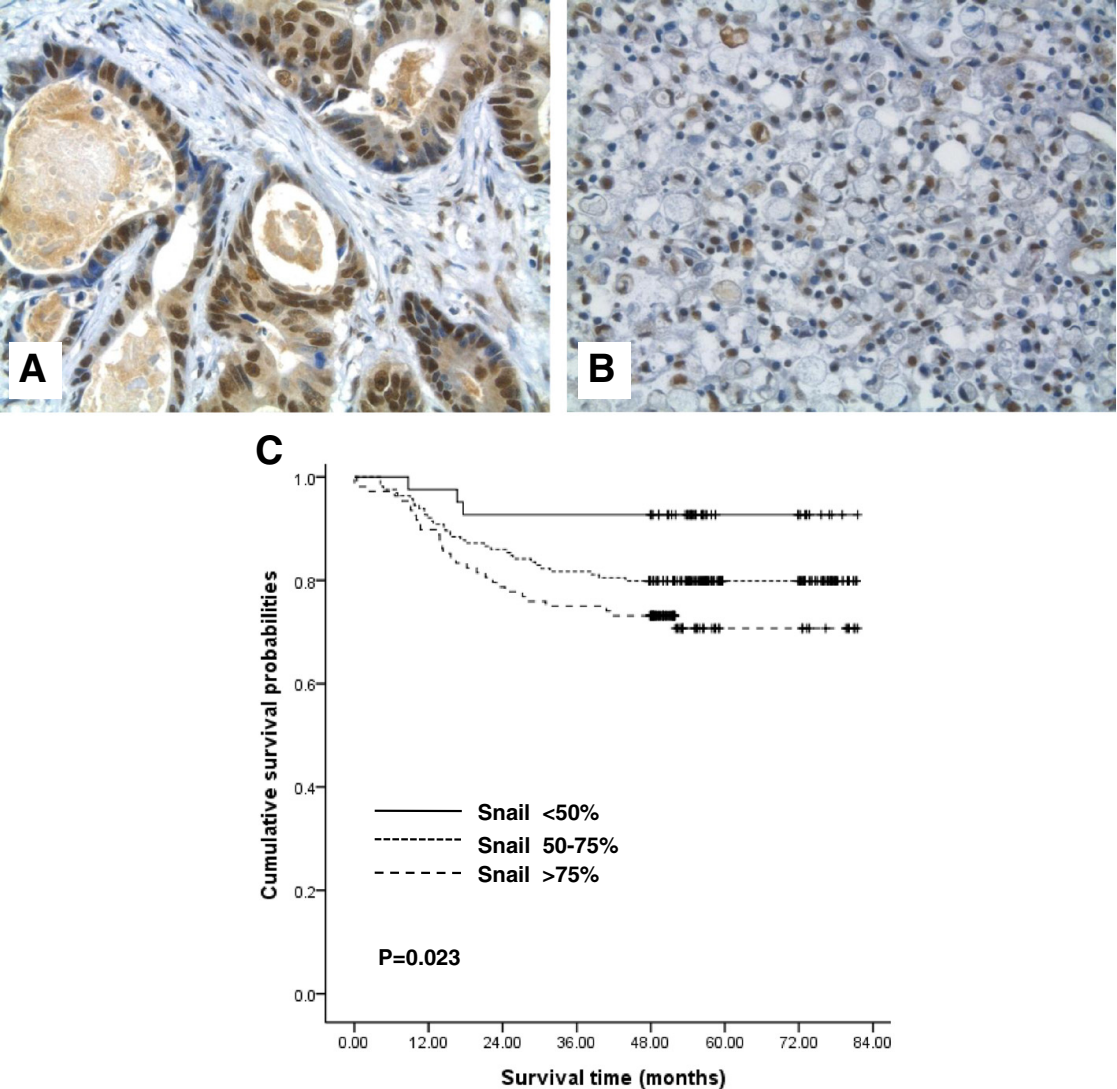
**Snail expression in gastric adenocarcinoma **(**GC**) **tissue samples and Kaplan**–**Meir plots of overall survival of 314 GC patients. **Snail was mostly expressed in nuclei of GC cells (intestinal type (**A**), and diffuse type (signet ring) cells (**B**)) included in tissue array specimens. Some reactive fibroblasts also showed Snail nuclear expression (magnification: ×400). **C**. Kaplan–Meier analysis of overall survival of GC patients based on Snail expression. A linear relationship between increased Snail nuclear expression and shorter survival was seen among GC patients (*P* = 0.023). Log-rank test was used to calculate *P* values.

Positive nuclear staining for Snail at levels of ≤50%, 50–75%, and ≥75% was observed in 13.4% (42/314), 52.2% (164/314), and 34.4% (108/314), respectively, of the 314 GC patients in immunohistochemical analysis. Snail expression was noted in intestinal and diffuse type of GCs (Figure 2A, B). Snail overexpression (≥75% positivity) significantly correlated with tumor size, gross type, depth of invasion, lymphovascular invasion, perineural invasion, and lymph node metastasis (Table 1). Snail overexpression was also associated with increased tumor size (*P* = 0.028) and excavated gross type (*P*< 0.001); and increased tumor invasiveness, i.e., higher T stage (*P*< 0.001) and the presence of perineural invasion (*P*< 0.001) and lymphovascular tumor emboli (*P* = 0.002). Increased lymph node metastasis was also related to Snail overexpression (*P* = 0.002).In accordance with the above data showing the positive relationship between Snail overexpression and GC aggressiveness, Snail overexpression significantly correlated with overall survival among GC patients (*P* = 0.023) (Figure 2C). A linear relationship was observed between increased nuclear expression of Snail and shortened survival (≤50%: 76.6 ± 2.7 months; 50–75%: 68.5 ± 2.0 months; ≥75%: 63.3 ± 2.8 months). Snail overexpression (≥75% positivity) was identified as an independent predictor of poor prognosis in 314 patients with GC, adjusted for age, sex, histologic classification, and tumor location, using a Cox regression proportional hazard model (*P* = 0.033; Table 2).

**Table 1:** Relationship between Snail expression and clinicopathological characteristics in 314 patients with gastric cancer

	**Number of patients (N = 314)**	**Snail Positivity**		***P *value**
		**<75%**	**≥75%**	
Age (years)		58.5 ± 10.6	59.1 ± 11.9	0.695
Sex				
Male	218	143	75	0.996
Female	96	63	33	
Tumor size				
≤4.0 cm	192	135	57	0.028
>4.0 cm	122	71	51	
Location				
Upper/Middle	167	112	55	0.561
Lower	147	94	53	
Invasion depth				
T1	160	127	33	< 0.001
T2	41	26	15	
T3	68	33	35	
T4	43	19	24	
Gross type				
Elevated	77	51	26	< 0.001
Flat/depressed	131	105	26	
Excavated	106	50	56	
Histological type				
Intestinal	182	123	59	0.609
Diffuse	122	76	46	
Mixed	10	7	3	
Perineural invasion				
Negative	202	150	52	< 0.001
Positive	111	55	56	
Lymphovascular emboli				
Negative	193	139	54	0.002
Positive	120	66	54	
Lymph node metastasis				
N0, N1	270	186	84	0.002
N2, N3	44	20	24	

**Table 2:** Multivariate survival analysis with Cox regression model in 314 gastric cancers

**Variables**	**B**	**SE**	**HR (95% CI)**	***P***
Age (≤59 versus > 59)	-0.438	0.264	0.645 (0.385-1.082)	0.097
Gender (male versus female)	-0.037	0.267	0.963 (0.571-1.626)	0.889
Site (upper and middle versus lower)	0.635	0.264	1.887 (1.126-3.164)	0.016
Lauren (intestinal vs diffuse)	-0.537	0.263	0.585 (0.349-0.978)	0.041
Snail (≥75% versus <75%)	-0.528	0.248	0.590 (0.363-0.958)	0.033

Note: B, coefficient; HR, hazard ratio; CI, confidence interval.

### Identification of gene expression patterns based on Snail overexpression using cDNA microarrays

**Figure 3:**
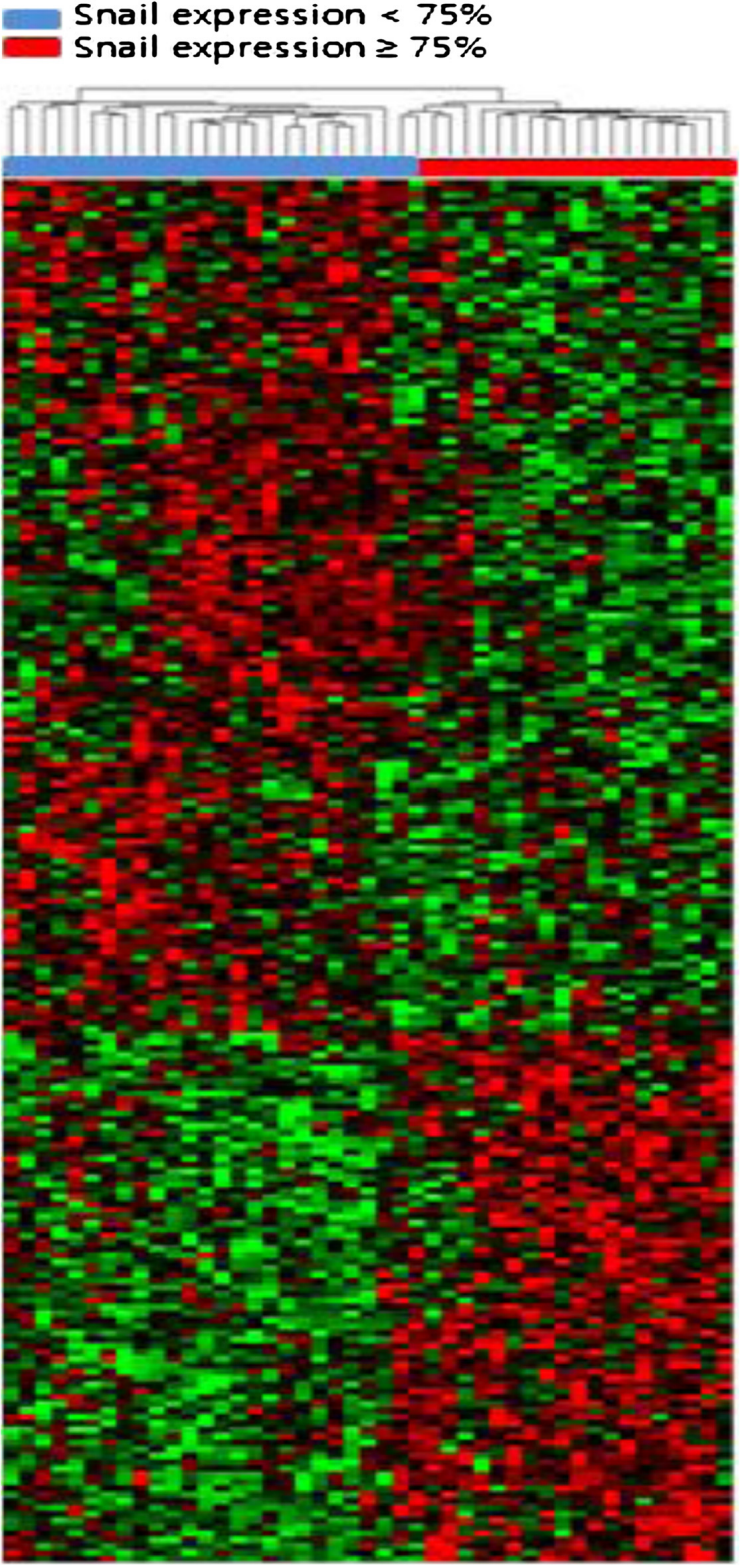
**Supervised clustering analysis of 45 gastric adenocarcinoma **(**GC**) **specimens and 172 genes. **Hierarchical clustering was used for 45 GC specimens and 213 genes. Data are shown in a matrix format, with rows representing individual genes and columns representing tissues. Each cell in the matrix represents the expression level of a gene featured in an individual tissue. Red and green cells reflect GCs with higher (≥75%) and lower (<75%) levels of Snail expression, respectively. Matrix patterns for specimens clustered into 2 distinct groups, except for one sample with higher levels of Snail expression.

cDNA microarrays were used to compare gene expression profiles of 45 GC specimens. We identified 213 genes that were differentially expressed at significant levels (*P* < 0.05) between GC specimens with higher (≥75%) and lower levels (<75%) of Snail expression (Table 3). Of these 213 genes, 82 were upregulated and 131 were downregulated in the GC specimens with higher levels (≥75%) of Snail expression. We used hierarchical clustering analysis to assess the 213 genes and 45 GC specimens; supervised clustering analysis gave patterns for samples with higher and lower levels of Snail expression clustered into 2 distinct groups, except for one sample with higher levels of Snail expression (Figure 3). To investigate the biological functions involved in discriminating genes, we performed a GO category analysis. Eleven genes were associated with regulating cancer cell–ECM adhesion (*P* < 0.021) and ECM protein regulation (*P* < 0.028, Table 4). Most have been implicated in cancer. *ONECUT1*, *ADAMTS*, *IFNAR2*, *MSR1*, and *SORL1* affect migration or metastasis, a process that involves attachment of tumor cells to the basement membrane, degradation of local connective tissue, and penetration and migration of tumor cells through stroma [21-25].

**Table 3:** Genes differentially expressed in GC specimens with higher levels of Snail expression

**PROBE**_**ID**	**SYMBOL**	**NAME**
Genes upregulated in specimens with higher levels (≥75%) of Snail expression (*P*< 0.05)		
ILMN_2374449	*SPP1*	Secreted phosphoprotein 1
ILMN_2337923	*TPD52L1*	Tumor protein D52-like 1
ILMN_1679838	*WBP5*	WW domain binding protein 5
ILMN_2078592	*C6orf105*	Androgen-dependent TFPI-regulating protein
ILMN_1714383	*TPD52L1*	Tumor protein D52-like 1
ILMN_1674817	*C1orf115*	Chromosome 1 open reading frame 115
ILMN_1813561	*SCIN*	Scinderin
ILMN_1759818	*SORL1*	Sortilin-related receptor, L(DLR class) A repeats containing
ILMN_1745686	*MFHAS1*	Malignant fibrous histiocytoma amplified sequence 1
ILMN_2060115	*SORL1*	Sortilin-related receptor, L(DLR class) A repeats containing
ILMN_2337263	*PKIB*	Protein kinase (cAMP-dependent, catalytic) inhibitor beta
ILMN_2173835	*FTHL3*	Ferritin, heavy polypeptide 1 pseudogene 3
ILMN_1791057	*IFNAR2*	Interferon (alpha, beta and omega) receptor 2
ILMN_1807114	*LOC255620*	Similar to unc-93 homolog B1 (C. elegans), transcript variant 1 (LOC255620), mRNA
ILMN_1669393	*GGT1*	Gamma-glutamyltransferase 1
ILMN_1685798	*MAGEA6*	Melanoma antigen family A, 6
ILMN_3269395	*GGT2*	Gamma-glutamyltransferase 2
ILMN_1669833	*SH2B2*	SH2B adaptor protein 2
ILMN_3238534	*LOC100133817*	Hypothetical protein LOC100133817
ILMN_2099315	*TRPM8*	Transient receptor potential cation channel, subfamily M, member 8
ILMN_3298065	*LOC729195*	Similar to apical early endosomal glycoprotein
ILMN_1717726	*FLJ43752*	Long intergenic non-protein coding RNA 336
ILMN_1670452	*ANKRD20A1*	Ankyrin repeat domain 20 family, member A1
ILMN_3201060	*LOC100132655*	Hypothetical protein LOC100132655
ILMN_3282829	*LOC727913*	Similar to iduronate 2-sulfatase (Hunter syndrome)
ILMN_2339691	*SYVN1*	Synovial apoptosis inhibitor 1, synoviolin
ILMN_1785549	*SLC30A2*	Solute carrier family 30 (zinc transporter), member 2
ILMN_3191898	*LOC100129630*	Hypothetical LOC100129630
ILMN_1704204	*LOC642204*	Ankyrin repeat domain-containing protein 26-like
ILMN_1682280	*LOC647753*	Hypothetical protein LOC647753
ILMN_3201944	*LOC646438*	Hypothetical LOC646438
ILMN_2233314	*SPANXA1*	Sperm protein associated with the nucleus, X-linked, family member A1
ILMN_3305980	*NS3BP*	NS3BP
ILMN_1747850	*CRIM2*	Kielin/chordin-like protein
ILMN_1700590	*LOC645590*	Similar to cAMP-dependent protein kinase type I-beta regulatory subunit
ILMN_1766316	*FLJ32679*	Golgin-like hypothetical protein LOC440321
ILMN_1890741	*Hs*.*552561*	Pancreatic islet cDNA clone hbt09690 3, mRNA sequence
ILMN_3308255	*MIR33A*	MicroRNA 33a
ILMN_1815716	*LMLN*	Leishmanolysin-like (metallopeptidase M8 family)
ILMN_1654945	*DNMT3A*	DNA (cytosine-5-)-methyltransferase 3 alpha
ILMN_2256050	*SERPINA1*	Serpin peptidase inhibitor, clade A (alpha-1 antiproteinase, antitrypsin), member 1
ILMN_1759487	*EGFLAM*	EGF-like, fibronectin type III and laminin G domains
ILMN_1760410	*LOC653086*	Similar to RAN-binding protein 2-like 1 isoform 2
ILMN_1668969	*MIXL1*	Mix paired-like homeobox
ILMN_3279757	*LOC100132532*	Hypothetical protein LOC100132532
ILMN_1715372	*CAMKK1*	Calcium/calmodulin-dependent protein kinase kinase 1, alpha
ILMN_1731370	*C9orf84*	Chromosome 9 open reading frame 84
ILMN_1679049	*COLEC12*	Collectin sub-family member 12
ILMN_1676011	*LOC642561*	Similar to FXYD domain-containing ion transport regulator 6
ILMN_1815442	*LOC652875*	Similar to Protein KIAA0685
ILMN_1737213	*LOC653641*	Golgin A6 family, member C
ILMN_1793529	*LOC389031*	Myosin
ILMN_1709319	*C13orf39*	Methyltransferase like 21C
ILMN_2284930	*FLJ40296*	Proline rich 20A
ILMN_1678310	*TXNRD3IT1*	Thioredoxinreductase 3 neighbor
ILMN_1806052	*UNC119*	unc-119 homolog (C. elegans)
ILMN_2242345	*LPAL2*	Lipoprotein, Lp(a)-like 2, pseudogene
ILMN_1687725	*C17orf41*	ATPase family, AAA domain containing 5
ILMN_1886395	*Hs*.*574341*	Soares_multiple_sclerosis_2NbHMSP *Homo sapiens*cDNA clone IMAGp998G11618; IMAGE:126826, mRNA sequence
ILMN_3308612	*MIR149*	MicroRNA 149
ILMN_1811103	*PCDHGB5*	Protocadherin gamma subfamily B, 5
ILMN_1736104	*LOC645218*	Hypothetical LOC645218
ILMN_1824307	*Hs*.*571901*	Full-length cDNA clone CS0DF20YK03 of Fetal brain of *Homo sapiens*
ILMN_1803871	*RHO*	Rhodopsin
ILMN_3237314	*LOC732402*	Similar to butyrate-induced transcript 1
ILMN_1714191	*LOC652682*	Similar to Y46G5A.1a
ILMN_3246580	*LOC730429*	e3 ubiquitin-protein ligase UBR5-like
ILMN_3229028	*LOC728586*	hCG1981531
ILMN_3239734	*LOC100134822*	Uncharacterized LOC100134822
ILMN_1769785	*SH3MD4*	SH3 domain containing ring finger 3
ILMN_3309864	*MIR449B*	MicroRNA 449b
ILMN_1653927	*SNORD83A*	small nucleolar RNA, C/D box 83A
ILMN_3200648	*LOC151174*	uncharacterized LOC151174
ILMN_1652023	*AGFG2*	ArfGAP with FG repeats 2
ILMN_1749776	*LOC642816*	Similar to hypothetical protein LOC284701
ILMN_1671985	*LOC646829*	Hypothetical protein LOC646829
ILMN_1684499	*LOC650373*	Similar to deubiquitinating enzyme 3
ILMN_1676452	*ADAMTS14*	ADAM metallopeptidase with thrombospondin type 1 motif, 14
ILMN_1723855	*LOC390427*	Similar to TBP-associated factor 15 isoform 1
ILMN_1658019	*LOC648447*	Hypothetical protein LOC648447
ILMN_3227291	*LOC728701*	Hypothetical LOC728701
ILMN_1767469	*LOC650781*	Hypothetical protein LOC650781
Genes downregulated in specimens with higher levels (≥75%) of Snail expression (*P*< 0.05)		
ILMN_1796946	*ALLC*	Allantoicase
ILMN_3248008	*LOC442308*	Tubulin, beta class I pseudogene
ILMN_3230623	*FLJ40039*	Uncharacterized LOC647662
ILMN_1676596	*LOC642263*	Hypothetical LOC642263
ILMN_3165745	*ERCC*-*00084*	Synthetic construct clone NISTag41 external RNA control sequence
ILMN_3242420	*HCG8*	HLA complex group 8
ILMN_1783827	*LOC649397*	Similar to Tripartite motif protein 44 (DIPB protein) (Mc7 protein)
ILMN_3244733	*LOC100131898*	Hypothetical protein LOC100131898
ILMN_3195376	*LOC100130092*	Similar to MAPRE1 protein
ILMN_2123683	*FLJ43763*	Uncharacterized LOC642316
ILMN_1730601	*FAM194A*	Family with sequence similarity 194, member A
ILMN_1652015	*LOC647451*	Similar to heat shock protein 90Bf
ILMN_1784349	*LOC647191*	Similar to Kinase suppressor of ras-1 (Kinase suppressor of ras) (mKSR1) (Hb protein)
ILMN_3251375	*WBP11P1*	WW domain binding protein 11 pseudogene 1
ILMN_1911713	*Hs*.*550068*	UI-E-EJ1-ajn-i-16-0-UI.s1 UI-E-EJ1 *Homo sapiens*cDNA clone UI-E-EJ1-ajn-i-16-0-UI.3, mRNA sequence
ILMN_1888057	*Hs*.*554470*	nc63e05.r1 NCI_CGAP_Pr1 *Homo sapiens*cDNA clone IMAGE:745952, mRNA sequence
ILMN_3229818	*LOC729828*	Misc_RNA (LOC729828), miscRNA
ILMN_1654987	*HCG2P7*	HLA complex group 2 pseudogene 7
ILMN_1683453	*FRAS1*	Fraser syndrome 1
ILMN_1840493	*Hs*.*112932*	ag03b01.s1 Soares_testis_NHT*Homo sapiens*cDNA clone IMAGE:1056169 3, mRNA sequence
ILMN_1860820	*Hs*.*126468*	tm27h01.x1 Soares_NFL_T_GBC_S1 *Homo sapiens*cDNA clone IMAGE:2157841 3, mRNA sequence
ILMN_3227213	*LOC728940*	Hypothetical LOC728940
ILMN_3247774	*LOC100134235*	Similar to hCG1642820
ILMN_1902571	*Hs*.*557622*	tw46h08.x1 NCI_CGAP_Ut1 *Homo sapiens*cDNA clone IMAGE:2262783 3 similar to contains PTR5.b2 PTR5 repetitive element, mRNA sequence
ILMN_2384405	*RTBDN*	Retbindin
ILMN_3234879	*LOC653786*	Otoancorinpseudogene
ILMN_1914891	*Hs*.*334272*	RST40254 Athersys RAGE Library *Homo sapiens*cDNA, mRNA sequence
ILMN_3272356	*LOC100129315*	Hypothetical protein LOC100129315 (LOC100129315), mRNA
ILMN_3230388	*LOC100130855*	Hypothetical protein LOC100130855( LOC100130855), mRNA
ILMN_1656553	*LOC653160*	Hypothetical protein LOC653160, transcript variant (LOC653160), mRNA
ILMN_1700935	*HAS2*	Hyaluronan synthase 2
ILMN_1733783	*LOC652790*	Similar to anaphase promoting complex subunit 1
ILMN_2209221	*DMRT1*	Doublesex and mab-3 related transcription factor 1
ILMN_1815118	*ZNF554*	Zinc finger protein 554
ILMN_3293210	*LOC100131031*	Similar to hCG2041190 (LOC100131031), mRNA
ILMN_1703222	*FRS2*	Fibroblast growth factor receptor substrate 2
ILMN_1732807	*GPRC6A*	G protein-coupled receptor, family C, group 6, member A
ILMN_1875332	*Hs*.*545527*	he15g04.x1 NCI_CML1 *Homo sapiens*cDNA clone IMAGE:2919216 3 similar to contains element PTR5 repetitive element
ILMN_3235789	*BPY2C*	Basic charge, Y-linked, 2C
ILMN_3203116	*LOC100131961*	Misc_RNA (LOC100131961), miscRNA
ILMN_2198802	*FAM22G*	Family with sequence similarity 22, member G
ILMN_1858700	*Hs*.*538558*	zh20c06.s1 Soares_pineal_gland_N3HPG *Homo sapiens*cDNA clone IMAGE:412618 3, mRNA sequence
ILMN_1873107	*Hs*.*282800*	AV649053 GLC *Homo sapiens*cDNA clone GLCBPH07 3, mRNA sequence
ILMN_1891673	*Hs*.*164254*	hb73c02.x1 NCI_CGAP_Ut2 *Homo sapiens*cDNA clone IMAGE:2888834 3, mRNA sequence
ILMN_3206632	*LOC643802*	u3 small nucleolarribonucleoprotein protein MPP10-like
ILMN_1883034	*Hs*.*546089*	RST29145 Athersys RAGE Library *Homo sapiens*cDNA, mRNA sequence
ILMN_2373335	*LIG3*	Ligase III, DNA, ATP-dependent
ILMN_3239639	*CD200R1L*	CD200 receptor 1-like
ILMN_1870857	*Hs*.*148168*	Barstead spleen HPLRB2 *Homo sapiens*cDNA clone IMAGp998L113601 ; IMAGE:1425178, mRNA sequence
ILMN_1813909	*CRSP2*	Mediator complex subunit 14
ILMN_1891885	*Hs*.*332843*	qg83a07.x1 Soares_NFL-T_GBC_S1 *Homo sapiens*cDNA clone IMAGE:1841748, mRNA sequence
ILMN_3235126	*LOC100133558*	Similar to hCG1642170
ILMN_1677186	*MGC52498*	Family with sequence similarity 159, member A
ILMN_3252608	*HCRP1*	Hepatocellular carcinoma-related HCRP1
ILMN_1652871	*PLSCR5*	Phospholipid scramblase family, member 5
ILMN_1698894	*OR5AS1*	Olfactory receptor, family 5, subfamily AS, member 1
ILMN_1705828	*RICTOR*	RPTOR independent companion of MTOR, complex 2
ILMN_1683046	*OR6Y1*	Olfactory receptor, family 6, subfamily Y, member 1
ILMN_2114812	*ONECUT1*	One cut homeobox 1
ILMN_1770248	*PDLIM2*	PDZ and LIM domain 2 (mystique)
ILMN_1784272	*CD1E*	CD1e molecule
ILMN_1755635	*FLJ33534*	Hypothetical protein FLJ33534 (FLJ33534), mRNA
ILMN_1799067	*TRY1*	Protease, serine, 1 (trypsin 1)
ILMN_1693448	*LOC643811*	Similar to FERM domain containing 6
ILMN_1723323	*HCG4*	HLA complex group 4 (non-protein coding)
ILMN_1865604	*Hs*.*253267*	60270330F1 NCI_CGAP_Skn3 *Homo sapiens*cDNA clone IMAGE:4800534 5, mRNA sequence
ILMN_3308698	*MIR1276*	MicroRNA 1276
ILMN_1714014	*LOC644491*	NMDA receptor regulated 2 pseudogene
ILMN_2114185	*C1orf104*	RUSC1 antisense RNA 1 (non-protein coding)
ILMN_1911044	*Hs*.*540915*	nf66b06.s1 NCI_CGAP_Co3 *Homo sapiens*cDNA clone IMAGE:924851 3, mRNA sequence
ILMN_1748543	*STRC*	Stereocilin
ILMN_1675221	*DGKZ*	Diacylglycerol kinase, zeta
ILMN_1726263	*LOC653748*	Similar to dipeptidylaminopeptidase-like protein 6 (dipeptidylpeptidase VI) (dipeptidylpeptidase 6) (dipeptidyl peptidase VI-like protein) (dipeptidylaminopeptidase-related protein) (DPPX)
ILMN_1817113	*Hs*.*547985*	UI-H-BI0p-abm-h-10-0-UI.s1 NCI_CGAP_Sub2 *Homo sapiens*cDNA clone IMAGE:2712450 3, mRNA sequence
ILMN_1793525	*KIR2DS3*	Killer cell immunoglobulin-like receptor, two domains, short cytoplasmic tail, 3
ILMN_2415617	*C10orf72*	V-set and transmembrane domain containing 4
ILMN_1746277	*MLLT4*	Myeloid/lymphoid or mixed-lineage leukemia (trithorax homolog, *Drosophila*); translocated to, 4
ILMN_1678246	*LOC644001*	Hypothetical protein LOC644001
ILMN_3257856	*LOC100130938*	Hypothetical LOC100130938 (LOC100130938), mRNA
ILMN_1865630	*Hs*.*116333*	Soares_testis_NHT*Homo sapiens*cDNA clone IMAGp998A031828, mRNA sequence
ILMN_2152028	*LOC642452*	Hypothetical LOC642452 (LOC642452), mRNA
ILMN_3244579	*LOC649330*	Heterogeneous nuclear ribonucleoprotein C-like
ILMN_1905832	*Hs*.*564127*	UI-E-DW1-ahc-g-05-0-UI.r1 UI-E-DW1 *Homo sapiens*cDNA clone UI-E-DW1-ahc-g-05-0-UI.5, mRNA sequence
ILMN_1897251	*Hs*.*547715*	UI-E-EJ0-ahv-e-11-0-UI.s1 UI-E-EJ0 *Homo sapiens*cDNA clone UI-E-EJ0-ahv-e-11-0-UI 3, mRNA sequence
ILMN_1782800	*LOC651410*	Hypothetical protein LOC651410
ILMN_1732554	*ZNF346*	Zinc finger protein 346
ILMN_1674014	*LOC653878*	Similar to Cytosolic acyl coenzyme A thioester hydrolase, inducible (Long chain acyl-CoA thioester hydrolase) (Long chain acyl-CoA hydrolase) (CTE-I) (CTE-Ib)
ILMN_1911501	*Hs*.*543905*	xi89f08.x1 NCI_CGAP_Mel3 *Homo sapiens*cDNA clone IMAGE:265999 3, mRNA sequence
ILMN_1878305	*Hs*.*262789*	xk07d09.x1 NCI_CGAP_Co20 *Homo sapiens*cDNA clone IMAGE:2666033 3, mRNA sequence
ILMN_1858245	*Hs*.*156566*	Soares_testis_NHT*Homo sapiens*cDNA clone IMAGp998M073519, mRNA sequence
ILMN_1704313	*GSTCD*	Glutathione S-transferase, C-terminal domain containing
ILMN_1707398	*ESRRB*	Estrogen-related receptor beta
ILMN_3307954	*L3MBTL4*	l(3)mbt-like 4 (Drosophila)
ILMN_1851244	*Hs*.*59368*	UI_H_BI1_aex-h-12-0-UI.s1 NCI_CGAP_Sub3 *Homo sapiens*cDNA clone IMAGE:2720903 3, mRNA
ILMN_1828556	*Hs*.*541581*	nac23e12.x1 Lupski_sciatic_nerve*Homo sapiens*cDNA clone IMAGE:3394270 3, mRNA sequence
ILMN_1692894	*LOC654042*	Similar to dehydrogenase/reductase (SDR family) member 4 like 2
ILMN_1893728	*Hs*.*377660*	*Homo sapiens*cDNA FLJ26242 fis, clone DMC00770
ILMN_1667005	*LOC652676*	Similar to similar to hypothetical protein FLJ36144
ILMN_3241607	*LOC100132106*	Hypothetical LOC100132106
ILMN_1797503	*GOLGA8G*	Golgin A8 family, member G
ILMN_1828034	*Hs*.*154513*	ik89c11.z1 Human insulinoma*Homo sapiens*cDNA clone IMAGE:6027645 3, mRNA sequence
ILMN_1886816	*Hs*.*544491*	qq31a07.x1 Soraes_NhHMPu_S1 *Homo sapiens*cDNA clone IMAGE:1934100 3, mRNA sequence
ILMN_1847950	*Hs*.*505398*	wq87c02.x1 NCI_CGAP_GC6 *Homo sapiens*cDNA clone IMAGE:2479010 3, mRNA sequence
ILMN_1734479	*ACCN3*	Acid-sensing (proton-gated) ion channel 3
ILMN_1675025	*H2BFM*	H2B histone family, member M
ILMN_2073279	*SIM1*	Single-minded homolog 1 (Drosophila)
ILMN_1910185	*Hs*.*98563*	zw57h03.s1 Soares_total_fetus_Nb2HF8_9w *Homo sapiens*cDNA clone IMAGE:774197 3, mRNA sequence
ILMN_3251491	*UQCRB*	Ubiquinol-cytochrome c reductase binding protein
ILMN_2180315	*ATG4D*	ATG4 autophagy related 4 homolog D (S. cerevisiae)
ILMN_1885583	*Hs*.*542934*	*Homo sapiens*cDNA FLJ26431 fis, clone KDN01390
ILMN_1743301	*MSR1*	Macrophage scavenger receptor 1
ILMN_1809820	*LOC648963*	Similar to retinitis pigmentosa 1-like 1
ILMN_1869348	*Hs*.*460114*	UI-E-EJ0-ahv-d-07-0-UI.s1 UI-E-EJ0 *Homo sapiens*cDNA clone UI-E-EJ0-ahv-d-07-0-UI 3, mRNA sequence
ILMN_1711332	*TFEC*	Transcription factor EC
ILMN_2228538	*IRAK1BP1*	Interleukin-1 receptor-associated kinase 1 binding protein 1
ILMN_1756455	*IL5RA*	Interleukin 5 receptor, alpha
ILMN_1719202	*ZNF174*	Zinc finger protein 174
ILMN_1847029	*Hs*.*553290*	HESC3_84_D06.g1_A036 Human embryonic stem cells *Homo sapiens*cDNA clone IMAGE:7483454 5, mRNA sequence
ILMN_1740217	*HACE1*	HECT domain and ankyrin repeat containing E3 ubiquitin protein ligase 1
ILMN_1787464	*LOC651296*	Similar to RAB, member of RAS oncogene family-like 2B isoform 1
ILMN_1734096	*DCLRE1A*	DNA cross-link repair 1A
ILMN_2391333	*CYP20A1*	Cytochrome P450, family 20, subfamily A, polypeptide 1
ILMN_2226314	*DBR1*	Debranching enzyme homolog 1 (S. cerevisiae)
ILMN_2379560	*CDC14B*	CDC14 cell division cycle 14 homolog B (S. cerevisiae)
ILMN_2078466	*DZIP1L*	DAZ interacting protein 1-like
ILMN_1653039	*LOC642934*	Hypothetical protein LOC642934 (LOC642934), mRNA
ILMN_2044293	*KBTBD7*	Kelch repeat and BTB (POZ) domain containing 7
ILMN_1809951	*ZNF200*	Zinc finger protein 200
ILMN_1760280	*NXT1*	NTF2-like export factor 1
ILMN_1657796	*STMN1*	Stathmin 1
ILMN_1793578	*ZFP37*	Zinc finger protein 37 homolog (mouse)

**Table 4:** Cellular functions of selected genes that are differentially expressed in GC specimens that overexpress Snail

**Probe ID**	**Gene acronym**	**Gene name**	**Accession No**.	***P *value**
Cancer cell–ECM adhesion				
ILMN_1759487	*EGFLAM*	EGF-like, fibronectin type III, and laminin G domains (↑)	NM_182801	0.005
ILMN_2114812	*ONECUT1*	One cut homeobox 1 (↓)	NM_004498	0.002
ILMN_2374449	*SPP1*	Secreted phosphoprotein 1 (↑)	NM_000582	0.004
ECM protein regulation				
ILMN_1676452	*ADAMTS14*	ADAM metallopeptidase with thrombospondin type 1 motif, 14 (↑)	NM_080722	0.005
ILMN_1759487	*EGFLAM*	EGF-like, fibronectin type III, and laminin G domains (↑)	NM_182801	0.005
ILMN_1683453	*FRAS1*	Fraser syndrome 1 (↓)	NM_020875	0.003
ILMN_1791057	*IFNAR2*	Interferon (alpha, beta, and omega) receptor 2 (↑)	NM_207585	0.001
ILMN_1756455	*IL5RA*	Interleukin 5 receptor, alpha (↓)	NM_000564	0.004
ILMN_1747850	*CRIM2*	Kielin/chordin-like protein (↑)	NM_199349	0.005
ILMN_1743301	*MSR1*	macrophage scavenger receptor 1 (↓)	NM_002445	0.002
ILMN_2374449	*SPP1*	secreted phosphoprotein 1 (↑)	NM_000582	0.004
ILMN_2256050	*SERPINA1*	Serpin peptidase inhibitor, clade A (alpha-1 antiproteinase, antitrypsin), member 1 (↑)	NM_000295	0.002
ILMN_2060115, ILMN_1759818	*SORL1*	Sortilin-related receptor, L(DLR class) A repeats-containing (↑)	NM_003105	0.003 <0.001

NOTE: ↑, upregulation; ↓, downregulation.

## Discussion

Snail is reportedly a key regulator of tumor progression and metastasis via increased MMP expression and tumor invasion [26,27]. Similarly, we found that upregulated Snail expression increased gastric cancer cell invasion/migration, whereas downregulated Snail expression decreased gastric cancer cell invasion/migration. Yang et al. reported that Snail overexpression in hepatocellular carcinoma cell lines induced increased invasiveness/metastasis [13]. In addition, Kosaka et al. reported that *Snail* knockdown was associated with decreased invasive capacity of a urothelial carcinoma cell line, supporting our results [12]. We also found that Snail overexpression induced increased expression of VEGF and MMP11, which are known markers of tumor invasion and metastasis. Jin et al. also reported that *Snail* knockdown by antisense *Snail* was associated with inhibited MMP activity, demonstrating the importance of regulating MMP activity in cancer metastasis.^10^ Furthermore, Peinado et al. reported that I MDCK cells with Snail overexpression had increased angiogenesis and VEGF [28]. We also observed increased VEGF in gastric cancer cells with Snail overexpression.

The clinical importance of Snail in various carcinomas, including non-small cell lung carcinomas, ovarian carcinomas, urothelial carcinomas, hepatocellular carcinoma, and breast cancer, is well known, as is the poor prognosis associated with Snail overexpression [10-13,29]. However, only limited immunohistochemical data have been available on Snail expression in GC, with no comprehensive clinical and functional analysis of Snail expression in GC patients. Kim et al. reported immunohistochemical data indicating that Snail expression was an independent indicator of prognosis in tissue microarray specimens [14]. Rye et al. reported that the combination of Snail, vimentin, E-cadherin, and CD44 was also significantly associated with poor prognosis in gastric cancer [15]. In contrast, no significant correlation between tumor stage and Snail expression was noted in upper gastrointestinal tract adenocarcinoma, including cancers of the esophagus, cardia, and stomach [30]. In our study, overexpression of Snail (≥75% nuclear Snail expression) was significantly associated with tumor progression, lymph node metastases, lymphovascular invasion, perineural invasion, and poor prognosis in GC patients. Recently, He et al. reported Snail to be an independent prognostic predictor of patient survival among gastric cancer patients; this is in agreement with our data [31]. Although 5-FU based adjuvant chemotherapy for advanced or metastatic gastric adenocarcinoma was usually performed in our cohort, further work is required to reveal exact significance of Snail expresssion as predictor of chemotherapy response in gastric adenocarcinoma. For the practical use of Snail as a tissue biomarker in predicting lymph node metastasis and poor prognosis, we defined a cut-off value of 75% positive nuclear expression for Snail overexpression. There are wide variations in cut-off values for Snail overexpression in different types of cancer; for example, 75% is used in non-small cell lung carcinoma [11], 100 (score of mean percentage × intensity, range 0–300) is used in urothelial carcinomas [12], and 50% is used in hepatocellular carcinoma [13]. For gastric cancers, cut-off values of 10% [14] and 5% [15] positive nuclear expression of Snail have been reported. Further work is required to determine a practical consensus cut-off value for Snail overexpression.

A total of 213 genes that were differentially expressed among GC samples with higher (≥75%) and lower levels of Snail expression were clustered into 2 distinct groups: those associated with regulation of cancer cell–ECM adhesion, and those associated with ECM protein regulation, such as *ONECUT1*[21], *ADAMTS*[22], *IFNAR2*[23], *MSR1*[24], and *SORL1*[25]. These functions indicate that Snail greatly affects cancer cell migration and metastasis by regulating attachment of tumor cells to basement membranes, degradation of local connective tissue, and penetration and migration of tumor cells through stroma.

## Conclusions

In this study, we showed that Snail overexpression induced increased migration and invasion in gastric cancer cell lines. Snail overexpression was also significantly associated with tumor progression, lymph node metastases, lymphovascular invasion, perineural invasion, and poor prognosis in GC patients. We identified 213 genes that were differentially expressed in GC tissues that overexpressed Snail, including genes related to metastasis and invasion by tumor cells. Our results indicate that Snail is crucial in controlling progression and metastasis of gastric cancer. Thus Snail may be used as a predictive biomarker for evaluating prognosis or aggressiveness of GCs.

## Competing interests

The authors declare that they have no competing interests.

## Authors’ contributions

NRS, EHJ, CIC and DYP were involved in the design of the study, collected the clinical data, performed the immunohistochemical analysis and drafted the manuscript. HJM performed *in vitro* study. CHK performed the analysis of microarray data and helped to draft the manuscript. ISC provided general support and helped to analyze the microarray data. GHK, TYJ, DHK and JHL provided the study materials or patients. DYP supervised the study. All authors read and approved the final manuscript.

## Pre-publication history

The pre-publication history for this paper can be accessed here:

http://www.biomedcentral.com/1471-2407/12/521/prepub
